# Perforated Gastric Ulcer With GI Bleeding Secondary to Cystic Artery Pseudoaneurysm

**DOI:** 10.1155/crra/2255883

**Published:** 2025-07-03

**Authors:** Akhil Tanwar, Surbhi Singh, Jennifer Hubert, Dhrumil Patel

**Affiliations:** Thomas Jefferson University Hospital, Philadelphia, Pennsylvania, USA

## Abstract

This case study presents an 87-year-old female patient with a history of chronic abdominal pain and NSAID use who was admitted with symptoms of hematemesis and melena, indicative of upper gastrointestinal bleeding. Upon examination, she was found to be hemodynamically stable but exhibited signs of moderate protein-calorie malnutrition. Imaging studies, including a multiphasic CT angiogram, revealed a contained rupture in the distal stomach, and a cystic artery pseudoaneurysm measuring 4.2 mm. Despite the presence of a perforated ulcer, there was no significant pneumoperitoneum or hemoperitoneum, leading to a diagnosis of contained perforation. The management plan included conservative treatment with IV antibiotics, proton pump inhibitors, and monitoring of hemodynamic status. On the third day of admission, the decision was made to embolize the cystic artery, as the risk of gallbladder ischemia was deemed low. This case underscores the critical need for prompt diagnosis and intervention in patients presenting with upper GI bleeding, particularly in the elderly, where the mortality rate can be significantly high. The findings emphasize the importance of imaging in localizing the source of bleeding and guiding appropriate management strategies.

## 1. Introduction and Case Presentation

An 87-year-old female with chronic abdominal pain presented to the emergency department with 1 day history of hematemesis and melena. In the ED, she experienced two episodes of hematemesis and a large volume melanotic stool followed by a syncopal episode.

On physical examination, her abdomen was soft and nontender. She had extreme nausea and was pale appearing and tachycardic. She had moderate protein–calorie malnutrition related to chronic illness. Hemoglobin was 8.6 g/dL and her guaiac stool test was also positive.

Her past medical history is positive for a right femur osteosarcoma complicated by hip disarticulation and right lower extremity amputation. She also has a long history of hypertension and primary osteoarthritis of the left knee. She did not have any recent imaging or any lab work. She had been taking NSAIDs for 1 month due to her chronic abdominal pain.

**Figure 1:**
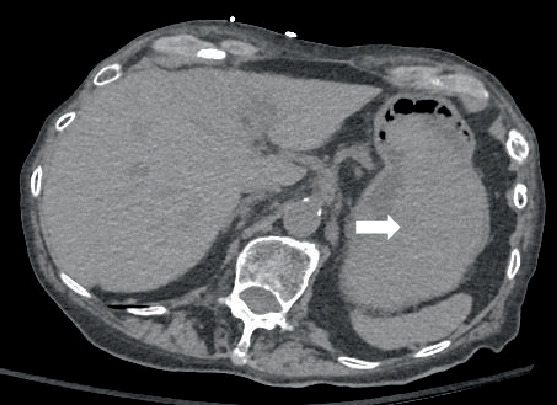
Noncontrast sequence shows hyperdense contents within the stomach that are most likely blood products (arrow).

**Figure 2:**
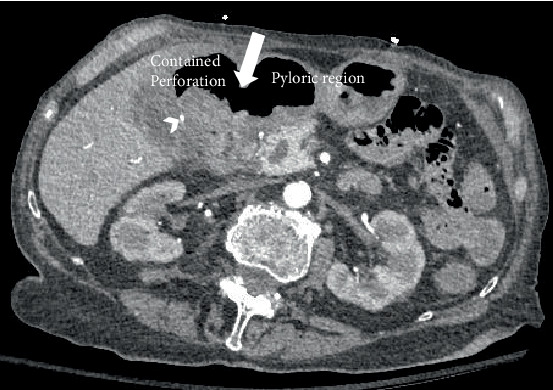
Arterial phase reveals a defect (arrow) along the anterior wall of the stomach in the antropyloric region. Contained perforation with hematoma. Pseudoaneurysm (chevron) of the cystic artery measuring up to 4 mm (no blush or active extravasation noted on the arterial phase).

Initially, a noncontrast CT abdomen pelvis was performed which showed hyperdense contents within the stomach, most likely representing blood products (Figure 1). Subsequently, a contrast CT was performed which revealed a defect/tear along the anterior wall of the stomach in the antropyloric region with extraluminal air and hematoma in the right upper quadrant. This was better delineated on the arterial and venous phases (Figure 2).

**Figure 3:**
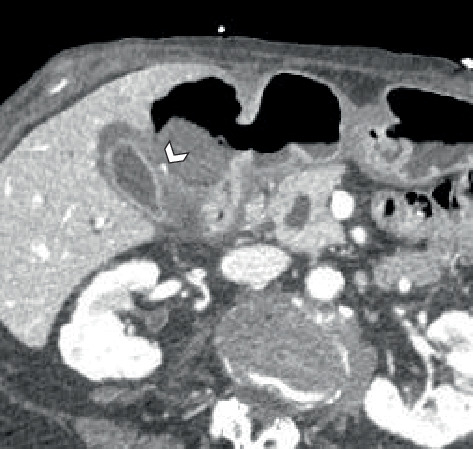
Venous phase reveals a small blush (chevron). Wall thickening of the GB. Dilated pancreatic duct, partially visualized.

Arterial phase showed a pseudoaneurysm of the cystic artery measuring 4 mm adjacent to the hematoma with no signs of active bleeding. A small blush was noted on the venous phase (Figure 3). Another ulcer was present along the posterior wall of the distal stomach.

**Figure 4:**
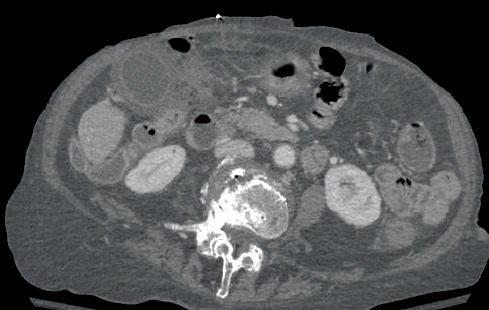
Marked GB wall thickening with inflammatory changes in the right upper quadrant.

**Figure 5:**
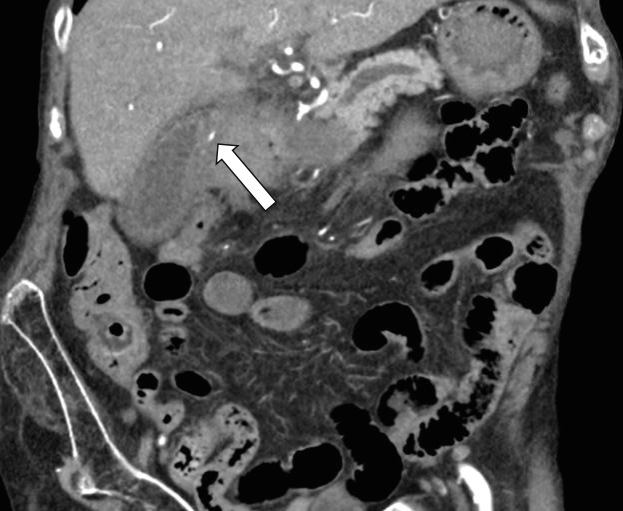
Arterial phase coronal view demonstrates the cystic artery aneurysm (arrow).

There was marked gallbladder wall thickening, which was favored to be secondary to inflammatory changes in the right upper quadrant and reactive in the setting of cystic artery pseudoaneurysm (Figure 4). There was no free air within the peritoneal cavity and no ascites/hemoperitoneum noted. Coronal reformats of the arterial phase also show the pseudoaneurysm without any extravasation (Figure 5).

Other nonacute findings included biliary ductal dilatation with mucosal enhancement concerning for cholangitis, enhancing 1.7 cm solid left renal lesion concerning for a possible malignancy, sigmoid diverticulosis, 7.6 cm right adnexal cyst and disarticulation of the right hip joint with right leg amputation. Calcified and noncalcified plaques in the abdominal aorta, right renal artery, bilateral common iliac, external and internal iliac arteries were also seen.

**Figure 6:**
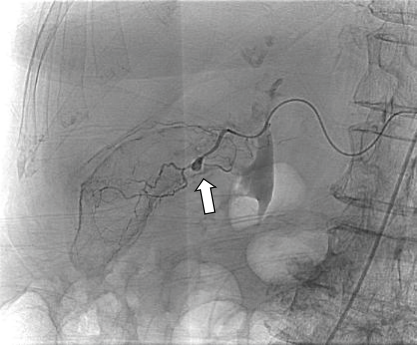
DSA shows a 4-mm saccular pseudoaneurysm of the cystic artery (arrow).

**Figure 7:**
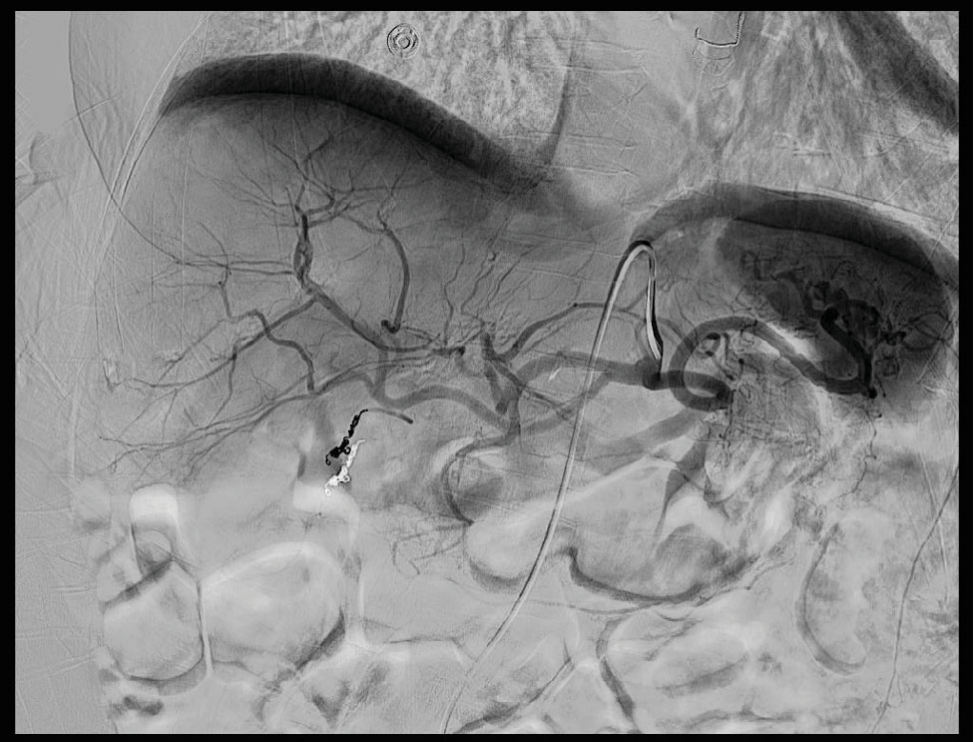
Successful postcoil embolization with no active contrast extravasation.

On the third day of hospitalization, cystic artery embolization was planned by the interventional radiology (IR). DSA demonstrated a 4-mm saccular pseudoaneurysm arising from the anterior cystic artery branch. No active bleeding was identified (Figure 6). Successful coil embolization with distal and proximal control across the anterior cystic arterial pseudoaneurysm was performed (Figure 7).

## 2. Discussion

According to PubMed, cystic artery pseudoaneurysm is a rare entity with only 67 cases reported between 1991 and 2020, and most were secondary to cholecystitis, with none due to gastric perforation. Other causes of cystic artery pseudoaneurysm include cholelithiasis and pancreatitis [1]. Pseudoaneurysm is a false aneurysm that occurs at the site of arterial injury, usually secondary to infection or trauma [2]. This is a unique presentation as the rupture or injury to the cystic artery vessel wall was likely caused by the perforation and “leakage/spill” of gastric contents in the subhepatic and pericholecystic region. The acidic juices likely damaged the vessel wall, resulting in the formation of a pseudoaneurysm.

Few classic symptoms of cystic artery pseudoaneurysm include hemobilia, jaundice, hypotension, right upper quadrant pain and swelling [3]. However, none of these classic sign and symptoms was present in our patient, hence the need for high index of suspicion. It could be argued that she might not have been experiencing any pain because she had been taking pain killers for the past 1 month.

Our patient, even though she had a perforated ulcer, did not reveal any significant pneumoperitoneum, ascites, or frank hemoperitoneum; hence, a “contained perforation” was the preliminary diagnosis. A study by Drakopoulos demonstrated that one can localize the site of perforation based on the amount of air and fluid in the abdomen [4].

Duodenal and gastric ulcers remain the two most common causes of perforations of the gastrointestinal tract due to the increased use of NSAIDS. [5] A contained gastric perforation occurs when there is a tear/defect in the wall of the stomach that is sealed off or ‘contained' by surrounding tissues, preventing the stomach contents such as ingested food material, gastric acid and enzymes from intraperitoneal spillage. Our patient likely developed a pyloric ulcer that subsequently perforated. Other causes for the development of peptic ulcers include *H. pylori* infection, hypovolemia, chronic steroid use, MEN syndrome, Zollinger–Ellison syndrome, smoking, and alcohol use. [6]

Our patient presented with upper abdominal pain, hematemesis, and melena, which are indicative of upper GI bleed (bleeding in the esophagus, stomach, or duodenum). Bleeding often results in a state of hypovolemia, which causes tachycardia. The guaiac test often detects blood in stool, supporting the possibility of ongoing GI bleed. Other causes of upper GI bleed are gastritis/erosive gastritis, esophageal varices, Dieulafoy lesion, gastric cancer, and Mallory–Weiss tear [7].

The next step in the management of the patient was to localize the site of the bleed and stabilize the patient. To localize the site of suspected bleeding, a multiphasic CT angiogram of the Abdomen was performed, which demonstrated two gastric ulcers, one of which demonstrated a contained rupture and associated right upper quadrant hematoma, likely resulting in the cystic artery pseudoaneurysm.

The key point of consideration in such a patient presenting with upper GI bleed is to promptly look for the cause of the hemorrhage as timely intervention is of utmost importance. Identifying contrast extravasation or vascular abnormality around the region of perforation would likely influence the treatment options for any patient.

Our patient was hemodynamically stable, and although IR consultation was obtained, no acute intervention was planned at the time; palliative care was offered. Since the patient was stable, she was treated with IV antibiotics and antifungals, proton pump inhibitors, and a decision was made to refrain from any surgical intervention and monitor hemodynamics and maintain hemoglobin > 8 mg/dL. In a study done in South Korea, a discharge hemoglobin of > 8 g/dL was linked to favorable outcomes [8].

Although, our patient did not have any acute complications, she had 1–2 bloody bowel movements overnight. On the third day of admission, decision was made to embolize the cystic artery. One of the main concerns was development of ischemic cholecystitis secondary to transcatheter arterial embolization of the cystic artery which was also reported in 50% of the patients in a retrospective study [9].

Our patient was deemed to be at a low risk for gallbladder ischemia given the short segment of artery embolized and perfusion of the remainder of the gallbladder via hepatic collateral vessels, but continued monitoring of hemodynamic status and abdominal exam was recommended. Successful postoperative recovery was noted.

## 3. Conclusion

In conclusion, this case highlights the complexity of managing upper gastrointestinal bleeding in elderly patients, especially those with comorbidities such as malnutrition and chronic NSAID use. Inflammation can result in formation of pseudoaneurysms and subsequent bleeding from the adjacent vasculature which may not be within the organ of origin. Scrutiny of not just the arterial phase but also venous/delayed phase is important for bleeds as often slow oozing is difficult to analyze on early arterial phase. The amount of pneumoperitoneum and ascites can often point to the site of perforation or can help localize the segment of bowel which should be carefully examined. Upper gastrointestinal bleeding is associated with high mortality in the elderly population; hence, a prompt and meticulous diagnosis by the radiologist can reduce complications and delay in interventional procedures.

## Data Availability

Data sharing is not applicable to this article as no datasets were generated or analyzed during the current study.
